# Protein and Gene Delivery Systems for Neurodegenerative Disorders: Where Do We Stand Today?

**DOI:** 10.3390/pharmaceutics14112425

**Published:** 2022-11-10

**Authors:** Panoraia I. Siafaka, Mehmet Evren Okur, Pelin Dilsiz Erim, Emre Şefik Çağlar, Emre Özgenç, Evren Gündoğdu, Rabia Edibe Parlar Köprülü, Ioannis D. Karantas, Neslihan Üstündağ Okur

**Affiliations:** 1 Faculty of Pharmacy, European University Cyprus, Nicosia 2404, Cyprus; 2 Department of Pharmacology, Faculty of Pharmacy, University of Health Sciences, Istanbul 34668, Turkey; 3 Department of Physiology, School of Medicine, Regenerative and Restorative Medical Research Center (REMER), Istanbul Medipol University, Istanbul 34810, Turkey; 4 Faculty of Pharmacy, Altınbaş University, Istanbul 34217, Turkey; 5 Department of Pharmaceutical Biotechnology, Faculty of Pharmacy, University of Health Sciences, Istanbul 34668, Turkey; 6 Department of Radiopharmacy, Faculty of Pharmacy, Ege University, Izmir 35040, Turkey; 7 Department of Medical Pharmacology, Institute of Health Sciences, İstanbul Medipol University, Istanbul 34810, Turkey; 8 Apostolos Loukas Medical Centre, Nicosia 2415, Cyprus; 9 Department of Pharmaceutical Technology, Faculty of Pharmacy, University of Health Sciences, Istanbul 34668, Turkey

**Keywords:** neurodegenerative diseases, genes, protein, monoclonal antibodies, peptides, theranostics

## Abstract

It has been estimated that every year, millions of people are affected by neurodegenerative disorders, which complicate their lives and their caregivers’ lives. To date, there has not been an approved pharmacological approach to provide the complete treatment of neurodegenerative disorders. The only available drugs may only relieve the symptoms or slow down the progression of the disease. The absence of any treatment is quite rational given that neurodegeneration occurs by the progressive loss of the function or structure of the nerve cells of the brain or the peripheral nervous system, which eventually leads to their death either by apoptosis or necrotic cell death. According to a recent study, even though adult brain cells are injured, they can revert to an embryonic state, which may help to restore their function. These interesting findings might open a new path for the development of more efficient therapeutic strategies to combat devastating neurodegenerative disorders. Gene and protein therapies have emerged as a rapidly growing field for various disorders, especially neurodegenerative diseases. Despite these promising therapies, the complete treatment of neurodegenerative disorders has not yet been achieved. Therefore, the aim of this review is to address the most up-to-date data for neurodegenerative diseases, but most importantly, to summarize the available delivery systems incorporating proteins, peptides, and genes that can potentially target such diseases and pass into the blood–brain barrier. The authors highlight the advancements, at present, on delivery based on the carrier, i.e., lipid, polymeric, and inorganic, as well as the recent studies on radiopharmaceutical theranostics.

## 1. Introduction

Protein and gene delivery targeting various diseases is a fascinating but complex and costly field of research. The use of genes and recombinant proteins, such as growth factors, and monoclonal antibodies has gained considerable interest in recent years since these molecules can pharmacologically act on genetic and metabolic disorders [11C]FLB 457 [1,2,3]. In particular, targeting neurodegenerative diseases (NDs) has been very challenging, given the nature of the diseases and the barriers that halt drugs’ entry into target tissues. Moreover, the main problem of neurodegenerative disorders is the underlying pathophysiological mechanism, which is still unknown. Despite the various advancements that have been made in recent years, the further study of early diagnosis and successful treatment should be performed.

For decades, patients suffering from neurodegenerative disorders have been treated with simple medications that focus on the symptoms’ relief and do not target the disorder. At present, various clinical trials evaluate drugs or other molecules, including antibodies, peptides, or hormones, which may target Alzheimer’s disease (AD) [4,5], Parkinson’s disease (PD) [6,7], and Huntington’s disease (HD) [8]. It seems that the updated knowledge of the possible underlying mechanisms has led to promising results for determining the root cause of these diseases, which could generate possible treatment strategies [9,10].

Gene- and protein-oriented therapeutic options primarily target the central nervous system (CNS), eye, or cochlea, implying that available active molecules cannot easily penetrate blood–brain barriers (BBBs), blood–cerebrospinal fluid barriers (BCSFBs), and blood–retina barriers (BRBs) [9,11,12,13]. Moreover, recombinant proteins, or gene therapies, are also large molecules that cannot cross the BBB, halting their use for Alzheimer’s disease (AD), Parkinson’s disease (PD), etc. It was not until 2021 when the first monoclonal antibody (MAb)-based drug (Aduhelm^®^) was approved by the Food and Drug Administration (FDA) as a therapeutic option for AD. However, the specific approval has been a controversial issue in the past year [14,15]. Beyond this authorization, the FDA has approved various MAbs for the management of other CNS or brain disorders, such as brain Bevacizumab (Avastin^®^) for brain cancer and Natalizumab (Tysabri^®^) for multiple sclerosis (MP). However, these MAbs are not able to penetrate the BBB and act by other mechanisms [16,17,18]. 

At present, there is a high demand to explore novel drug agents and focus on biologic molecules that are less toxic and present a higher safety profile to target NDs. Furthermore, it is important to identify novel delivery routes that can provide better penetration of the BBB or BCSFB through the intranasal route [19] via, for example, subpial injections [20]. The conventional delivery routes cannot actually be used for the delivery of biological molecules since their half-life and degradation by gastric fluids can also result in a low therapeutic effect [21]. Intravenous delivery cannot be applied for the delivery of biologics since the drugs can rarely surpass the BBB, while intramuscular injections or subcutaneous delivery might be an alternative option to efficiently cross the physiological barriers with clinical benefits [9,22].

Therefore, the future and present therapeutic strategies of ND should combine disease targeting, the penetration of the possible barriers, with as few side-effects as possible. In this review, a summary of the recent advancements in protein and peptide delivery systems to target neurodegenerative disorders, such as AD, PD, and Huntington’s disease (HD), are attempted. Various articles about the pharmaceutical formulations to date, such as nanotechnology-based systems for the delivery of proteins and peptides, are discussed. They are categorized according to the materials used, i.e., polymers, lipids, or inorganic nanoparticles. Most of the categories incorporate the use of polymers, since they are the most versatile materials. In addition, the neurodegeneration mechanisms and disorders of AD, PD, HD are examined. The article’s focus is to assist any student or healthcare worker to broaden their knowledge of this complex issue.

## 2. Neurodegenerative Disorders and Their Underlying Mechanisms

### 2.1. Mechanisms of Neurodegeneration

**Figure 1:**
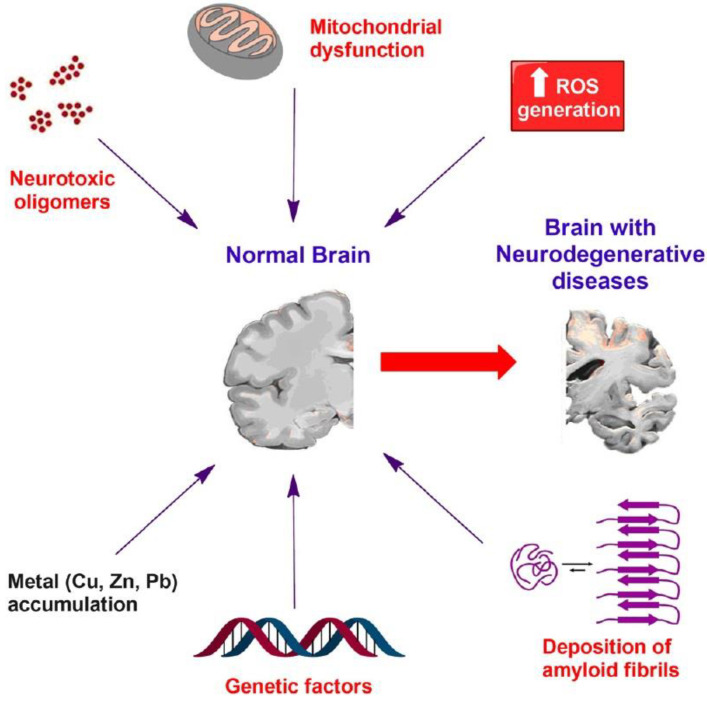
The multifactorial and complex nature of neurodegenerative diseases. Various mechanisms of neurodegeneration are proposed i.e., metal accumulation, neurotoxic oligomers, mitochondrial dysfunction, genetic factors, ROS (reactive oxygen species), and deposition of amyloid fibrils [25]. Reprinted from Ibrahim and Gabr. Copyright © 2019 Neural Regeneration Research was published by Wolters Kluwer Medknow Publications under Creative Commons Attribution-NonCommercial-ShareAlike License (CC BY-NC-SA).

The majority of mechanisms that are responsible for neurodegenerative diseases include genetic, environmental, endogenous, or epigenetic factors, along with their multifactorial combinations [23]. The basic categorization of neurodegeneration mechanisms focuses on protein dynamics, such as abnormal protein aggregation on the intracellular or extracellular level, misfolding, degradation, or dysfunction of proteasomes. Other mechanisms include increased oxidative stress, mitochondrial dysfunction, abnormal cellular fragmentation, impaired neuronal transport, and neuroinflammation [24]. In this section, the pathogenic mechanisms underlying neurodegenerative diseases are discussed (Figure 1). 

#### 2.1.1. Disrupted Protein Dynamics

In most of the neurodegenerative diseases, protein dynamics are significantly altered. When disrupted, protein interactions cause the aberrant aggregation, oligomerization, or misfolding of disease-specific proteins, which in turn cause cellular abnormalities, synaptic impairment, and brain damage, which are symptoms commonly observed in a wide range of neurodegenerative diseases [26,27]. The aggregated proteins target specific neuron populations, albeit the same neuronal groups might be influenced by diverse NDDs [28], resulting in neuronal and glial cell death or the disruption of neural circuitry [29]. Proteins aggregate into different NDDs and vary significantly in protein sequence, structure, and function along with the level of expression. These proteins, on the other hand, exhibit a similar misfolding of intermolecular β-sheets, which then accumulate to form fibrillar structures in either highly ordered or amorphous forms [30]. The mechanism of disrupted protein dynamics observed in neurodegenerative diseases is best described by the seeding-nucleation model. In this model, first the protein nucleus, or in other words the seed, is formed, and then the polymerization of protein monomers is triggered, which then gives rise to more seeds. Subsequently, these seeds attract more proteins and initiate protein aggregation [31,32]. The aggregation process includes the arrangement of proteins into β-pleated sheets with hydrogen bonding, which recruits either folded or semi-folded proteins and initiates their misfolding process in order to include them in the β-sheet structure [26]. The misfolding of normal and functional proteins initiates the accumulation. Improper protein folding is not the sole reason for aggregation. The disrupted protein interaction and production, overexpression of the protein, weak protein turnover, changes in post-translational modifications, oxidation of proteins, improper proteolysis, abnormal gene splicing, and impaired activity of molecular chaperons are among the reasons for intracellular protein aggregation [24]. Protein folding is essential to provide a physiological function to native proteins, and it is directed by ubiquitous molecular chaperons in order to avoid an abnormal interaction or misfolding due to cellular stress [33]. In their native forms, chaperones, such as heat-shock proteins (Hsp), promote normal protein folding and recovery from protein damage. For instance, Hsp40 and Hsp70 induce NDD turnover by the ubiquitin–proteasome system. Hsp90, on the other hand, regulates misfolded proteins that cause NDDs [34]. However, the mutations observed in molecular chaperones cause neurodegeneration, which in turn trigger the aggregation of proteins involved in cell survival signal transduction pathways and induce NDDs, such as Parkinson’s and Huntington’s diseases [33,35,36]. 

#### 2.1.2. Mitochondrial Dysfunction

Normal neuronal or glial cell function depends on extended ATP production, which enables neural communication. There is growing evidence suggesting that impaired mitochondrial homeostasis plays a critical role in neurodegeneration [37]. For example, in Alzheimer’s disease, disrupted mitochondrial dynamics, which includes alterations in mitochondrial morphology, bioenergy, or transport, are responsible for synaptic degeneration and the aggregation of neurofibrillary tangles, along with Aβ accumulation, which is known to affect mitochondrial dynamics, including fusion that coordinates mitochondrial enzymes and ceases mitochondrial aging and fission that facilitates autophagy [38,39]. 

### 2.2. Classification of Neurodegenerative Disorders

**Figure 2:**
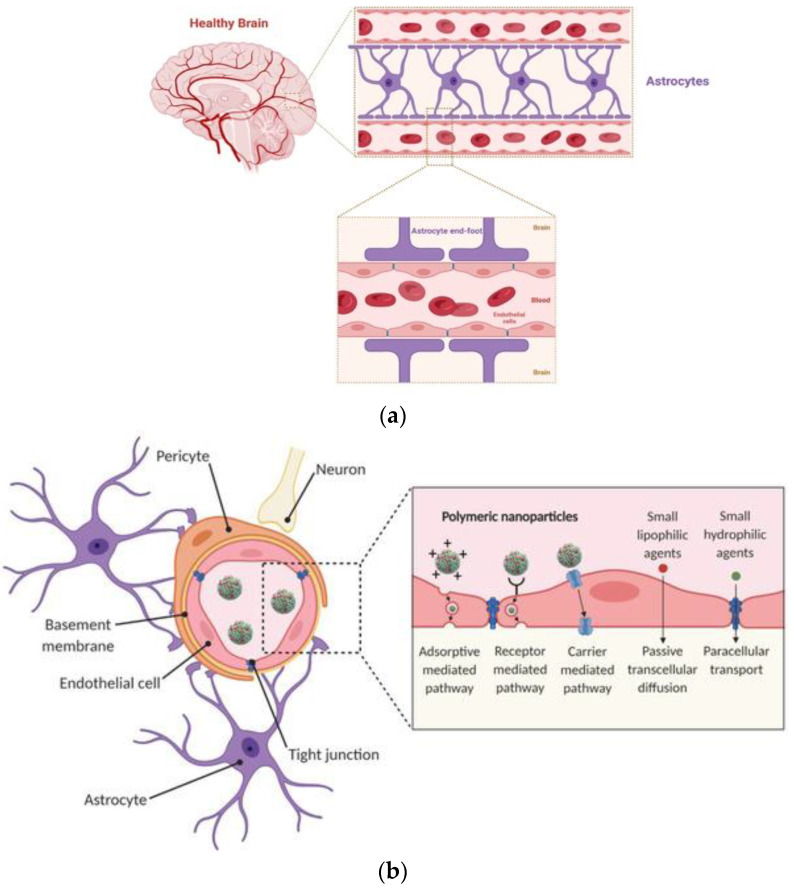
(**a**) Anatomy of blood–brain barrier (created with BioRender.com) and (**b**) transportation of polymeric nanoparticles to the brain via various pathways. Reprinted from Zhang et al. [42]. Copyright © 2021 Advanced Science published by Wiley-VCH GmbH under Creative Common License 4.0.

Every year, neurodegenerative disorders affect millions of people; it has been estimated that at least 55 million people live with dementia [40]. In Alzheimer’s or Parkinson’s diseases, neurons are damaged or affected, resulting in their death and the progression of the disease. The human brain and its 86 billion neurons need an adequate blood supply to function, which is achieved via an extensive, well-ordered vascular network composed of arteries, arterioles, capillaries, venules, and veins [41]. The BBB is composed of a continuous endothelial monolayer that maintains cerebrovascular integrity between the endothelium, wall cells, astrocytes, and neurons. The BBB (Figure 2a) regulates the entrance of blood-derived macromolecules, blood cells (e.g., leukocytes), as well as microbes to the brain, whereas it is found at all levels of the vascular tree [41]. Small molecules, drugs, and polymeric nanoparticles can penetrate the BBB via various pathways. Polymeric nanoparticles, for example, travel across the BBB via carrier-mediated, receptor-mediated, and adsorptive-mediated pathways [42] (Figure 2b). Neurodegenerative diseases (NDs) occur form the damage to the central and peripheral nervous systems. Neurological disorders might occur due to ischemia, cellular stress, neural tissue inflammation, neuronal loss due to autoimmunity, or infection. At present, most treatments focus on temporary symptomatic relief [11,43].

**Figure 3:**
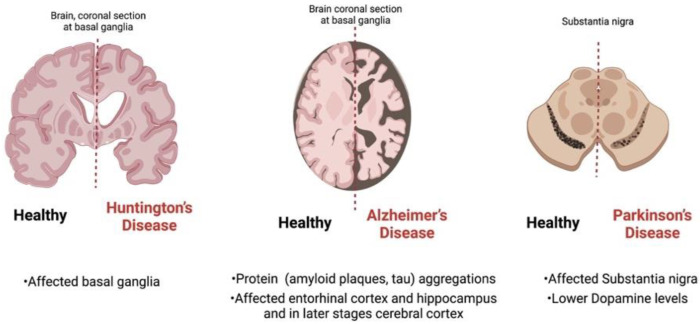
Main observations of a patient’s brain with neurodegenerative disorders. HD affects the basal ganglia, resulting in affective, cognitive, behavioral, and motor decline. AD affects the hippocampus, entorhinal cortex, and cerebral cortex, leading to brain shrinkage, memory loss, and general and thinking skills decline. PD, which affects the substantia nigra, and low dopamine levels cause bradykinesia, tremors, and motor impairment (created with BioRender.com).

During brain aging, various metabolic, morphological, and neurophysiological alterations occur, along with impaired learning and memory deficits. Furthermore, consequent synaptic loss, declined N-Methyl-D-aspartate (NMDA)-receptor responses, as well as altered calcium homeostasis have also been recorded [44]. It has been reported that aging has been linked to obesity, metabolic syndrome, and neurodegenerative diseases, and thus their prevalence has increased [44]. Obesity has been associated with decreased brain volumes [45], gray-matter density [46], and cognitive function [47]. Subjective cognitive complaints as well as mild cognitive impairment are common among patients with PD, while the first clinical manifestation of AD is also mild cognitive impairments. Such changes can be observed, even in middle-aged patients, along with other psychiatric or behavioral symptoms [48]. According to the National Institute on Aging-Alzheimer’s Association, clinicians who diagnose dementia should be aware of changes in personality and behavior [49]. For example, social exclusion and not being able to handle emotions could be signs of a problem with social cognition [50]. Furthermore, sleep disturbances, such as insomnia, apnea, restless-legs syndrome, and an abnormal circadian rhythm, could be underlying symptoms of a neurodegenerative disorder in older adults. Therefore, the clinical symptomatology should be identified by the clinician and managed, since treatment can improve the neurodegenerative condition and quality of life of the patient [48]. Neurodegenerative disorders are mainly categorized according to their clinical manifestations; the most common symptoms are extrapyramidal and pyramidal movement disorders, as well as cognitive or behavioral disorders [51]. Therefore, AD leads to memory loss or personality alterations; PD leads to an impaired motor capacity and attention deficit; and amyotrophic lateral sclerosis leads to weakness and cognitive decline [43]. Furthermore, the aforesaid disorders have been specifically described as neuronal cell loss and abnormal protein deposition in brain cells. Typically, neurodegenerative disorders are defined by the deposition of specific protein deposits and anatomical fragility. However, a progression of neuronal dysfunction and death, including proteotoxic stress, abnormalities in the ubiquitin–proteasomal system, neuroinflammation, and oxidative stress, has also been identified by experts [51]. Figure 3 summarizes the basic observations of a patient’s brain with neurodegenerative disorders.

#### 2.2.1. AD and Dementia

Dementia has been linked to various causes and a variety of symptoms [52]. In mixed dementia, AD, and vascular dementia, in consideration of similar clinical manifestations, their diagnosis as medical entities is very difficult [53]. Normally, the basic symptoms of dementia are memory loss, language difficulties, problem-solving difficulties, as well as other cognitive skills that may affect the capability of a patient to perform daily activities. It has been reported that the symptoms occur due to damage to or destruction of neurons in brain areas related to cognitive function [54]. Almost 10 million European citizens have been diagnosed with dementia, and the majority of them belong to the Alzheimer’s spectrum [53]. It is believed that over the next 50 years, the number of people living in Europe who are over 65 years of age and will develop dementia will double from 87 million in 2010 to 148 million in 2060 [53]. 

AD is the most frequent form of disabling degenerative dementia in the elderly, affecting more than 50 million people worldwide [54,55]. It is characterized by reduced cognitive functions as well as a loss of memory [52]. It progresses over time, which eventually leads to a decline in life quality and death [52,56]. The economic burden of AD is a consideration of significant mortality and morbidity rates [57]. Due to the numbers, the World Health Organization has recognized AD as a global public health priority [58]. The clinical manifestations of AD involve both cognitive impairment and progressive neuropsychiatric or behavioral symptoms, causing functional disability [59]. A significant risk factor for AD is the cerebral vascular pathology, which has been linked to lower scores in most cognitive domains. Furthermore, small-vessel brain disease is notable in AD sufferers and accounts for half of all dementia diagnoses worldwide [60]. Some researchers have indicated that many patients with dementia symptoms and that present brain abnormalities are related to more than one dementia cause [54]. 

AD is a progressive neurodegenerative disorder, triggering the degeneration and eventual death of brain cells, including those that allow patients to perform daily functions, such as walking and swallowing [52]. The pathophysiological mechanism of Alzheimer’s disease is unknown; however, neurofibrillary tangles composed of amyloid- β (Aβ) peptides and hyperphosphorylated tau are some of its indicators [54]. For example, Alzheimer’s disease (AD) is distinguished by the presence of intracellular neurofibrillary protein aggregates known as neurofibrillary tangles (NFTs), which are composed of tau proteins, as well as extracellular β-amyloid (Aβ) accumulation that results in hyperphosphorylated, prion-like misfolded oligomers [44]. Aß peptides seem to be important keystones for brain degenerative alterations and appear due to the enzymatic cleavage of the amyloid precursor protein (APP) [53]. Amyloid oligomers can also disrupt the integrity of cell membranes, leading to an unbalanced flow of extracellular Ca^2+^, altering homeostasis [44]. Furthermore, AD progression has been linked to the extracellular accumulation of Aβ plaques, intracellular tau-protein inclusions in neurofibrillary tangles, as well as the degeneration of neurons [53]. Furthermore, mutations in the APP and secretase enzymes play a role during APP processing and could lead to high amounts of Aβ, and eventually large fibrillar deposition [44]. AD seems to deactivate APP, presenilin-1 (PSEN1), and presenilin-2, presenting a complex genetic architecture with rare, highly penetrating mutations in the genes coding for (PSEN2) [61]. ß-amyloid peptides, which comprise amino acids 36–43, are the main elements of amyloid plaques that have been found in the brains of AD patients; consequently, a major therapy targeting AD management is Aβ targeting [53]. Another interesting point is that mitochondrial dysfunction has been linked to damaged neurons in both sporadic and familial types of the disease, as well as in animal models of AD. Therefore, mitochondrial accumulations are key pathological features of AD [54]. Some studies report that mitochondrial dysfunction speeds up Aβ production before the accumulation of Aβ deposits in the brains of AD mouse models, which is important for the AD process [54].

Another possible explanation for the presence of AD neurodegeneration is that the histopathological, molecular, and biochemical abnormalities associated with decreased glucose utilization during the disease could point to a link between insulin metabolism and the clinical manifestation of AD [44]. Among others, inflammatory processes as well as other alterations of the immune system have been ascribed to AD. It seems that individuals who can benefit from Aβ-lowering drugs should be in the early stages of the disease, where extensive brain damage has not yet occurred. Moreover, given that AD pathology is not reversible, disease-modifying medication can only be helpful during the early diagnosis of AD. Patients in the advanced stages of Alzheimer’s disease, on the other hand, may experience a significant improvement in their symptoms if disease progression is slowed down or even stopped [53]. The management therapies of AD include cholinergic therapy, anti-glutamatergic therapy, vitamins and antioxidants, nonsteroidal anti-inflammatory drugs (NSAIDs), and the pharmacological treatment of neuropsychiatric symptoms [62].

Despite the low success rate of AD drug development, the recent approvals of gene or protein-based therapeutics have changed the landscapes of pharmaceutical development and therapy for AD. As the molecular understanding of AD biology and its adjustment in treatment increases, combination therapy is to be expected as a treatment option. Additionally, the likelihood of effective drug development increases with better demographics and trial designs. A patient’s characteristics, such as age, gender, genetic factors, medical issues, environmental effects, and lifestyle, are all taken into account while determining a course of therapy. Machine learning developments have made it possible for us to deal with complex elements and develop models that predict the most effective therapy options for AD patients. Further research is required to establish the relationship between a patient’s characteristics and therapy response. With increasing research efforts, precision medicine is a practice that is both anticipated and practicable [63,64].

#### 2.2.2. Parkinson’s Disease (PD) 

Parkinson’s disease (PD) is the second most common neurodegenerative disorder after AD and is defined as a progressive movement disorder [65,66,67]. It is reported as a central nervous system disease attributed to the chronic and progressive neurodegeneration of the substantia nigra that produces dopamine (DA); DA is essential for the circuit activation that controls movement [44]. The clinical manifestations of PD include non-motor symptoms, such as tremors, bradykinesia [68], rigidity [69], instability [70], sleep, and mood disturbances [65]. The economic burden of PD is increasing; it is estimated that its global prevalence is expected to double from 6.2 million cases in 2015 to 12.9 million cases by 2040 [67]. Although the incidence of PD in patients younger than 50 years old is lower, it is believed that Parkinson’s disease in humans affects approximately 1% of people older than 60 years of age annually, worldwide [65]. The most common risk factor of PD is advancing age [66], although both the environment and genetics [70] can influence disease risk and progression [44]. For example, a potential link between PD and several environmental characteristics, such as pesticide exposure [70], smoking [71], and caffeine intake [44], has been reported. Family history and genetics are also risk factors for Parkinson’s disease [55,72]. Even though PD is a disease that worsens over time, it is one of the few neurodegenerative diseases where the symptoms are easy to manage with dopamine-replacement therapy [65]. 

PD is pathologically characterized by a loss of dopaminergic neurons in the substantia nigra (SN) pars compacta (SNpc), and the presence of Lewy bodies accumulating in the midbrain [66]. Lewy bodies are intraneuronal, round, eosinophilic inclusions with a hyaline nucleus and a pale peripheral halo comprising various proteins, such as α-synuclein (encoded by the SNCA gene) [67]. Lewy bodies, which consist of α-synuclein and ubiquitin, tend to misfold, become insoluble, and form b-layer-rich amyloid aggregates that accumulate and form intracellular inclusions [72]. During this aggregation process, the toxic oligomeric and proto-fibrillar forms impede mitochondrial, lysosomal, and proteasomal functions, harm biological membranes and the cytoskeleton, change synaptic functions, and finally cause neuronal degeneration [72]. Dopamine-secreting neurons dying off can throw off the balance of excitatory (acetylcholine) and calming (dopamine) neurotransmitters in the area, causing uncontrollable behavior, such as dyskinesia and freezing of the gait [68]. 

PD is usually clinically diagnosed based on its signs and symptoms [68], including tremor syndromes, such as essential tremors, atypical Parkinsonism, as well as other tremor disorders, secondary Parkinsonism [72], and other cognitive disorders. Furthermore, it is very common to observe a sustained response to dopamine drugs (dopamine agonists or levodopa) [68]. Moreover, two main neuropathologies, such as the loss of pigmented dopaminergic neurons in the substantia nigra or the buildup of α -synuclein in neurons, can be used together to perform a definitive diagnosis of idiopathic Parkinson’s disease [71]. The medical management of PD includes symptomatic relief [68] and a decrease in dyskinesia by predominantly focusing on the dopaminergic pathway [72]. Pharmacological approaches are initiated when symptoms cause disabilities [68]. In the early stages of PD, tremors can be relieved by beta-blockers, primarily by propranolol [68]. Furthermore, some motor symptoms are caused by a lack of dopamine; thus, levodopa is the gold-standard drug used for motor-symptom relief in the treatment of Parkinson’s disease [72]. It crosses the BBB, converting to dopamine in the remaining dopaminergic neurons of the SNPC. The therapeutic potential of MAO-B inhibitors, such as Rasagiline and selegiline, is based on their ability to reduce dopamine metabolism by prolonging and enhancing dopaminergic stimulation [72].

The Parkinson’s Foundation estimates that approximately 10 million people worldwide have the disease, and that PD incidence rises with advancing age. Each patient’s PD disease development and symptoms are distinct from one another, and each patient’s treatment is chosen based on these factors. Some of the experimental therapies that target the pathological hallmark of a-synuclein accumulation include small molecule inhibitors of a-synuclein accumulation, passive immunization with monoclonal antibodies, and active immunization with immunogenic peptides that produce antibodies to a-synuclein. The potential neuroprotective effects of C-Abelson kinase (c-Abl) inhibitors have been studied; however, nilotinib presents conflicting results due to its poor central nervous system penetration; therefore, other possibilities are being researched. Even though many of these initially attractive paths have failed in larger studies, there is still a plan that will eventually lead to meaningful medication and disease change for PD. Despite the fact that COVID-19 posed a substantial challenge in 2021–2022, considerable advancements in the clinical development of symptomatic treatments (STs) and disease-modifying therapeutics (DMTs) for Parkinson’s disease (PD) have nonetheless been made. Throughout the time period, the total number of trials and the distribution of STs and DMTs remained constant. While numerous projects have been removed from the list, it seems that progress toward Phase 1 and from Phase 1 to Phase 2 is still being made. The lack of progression of DMTs from Phase 2 to Phase 3 is still a cause for concern and is a significant rate-limiting stage in the development of novel treatments for persons with Parkinson’s disease. However, 13 DMT Phase 2 trials are expected to be completed by the end of 2022, and several Phase 3 trials are already in the planning stage. In the upcoming years, there will hopefully be further DMT Phase 3 trials as a result of this. Future research is anticipated to result in the expansion of our treatment options and the identification of unique predictors for the most effective treatment approaches [73,74,75]. 

#### 2.2.3. Huntington’s Disease (HD)

Huntington’s disease (HD) is a neurodegenerative disorder caused by a hereditary CAG trinucleotide re-expansion [8] of the Huntington’s gene on chromosome pair number 4 [44]. It affects the central nervous system, [44] inducing progressive neuronal damage, mainly in the basal ganglia and cerebral cortex [8,76]. HD has a prevalence of 10.6 to 13.7 people per 100,000 in Western populations [8]. HD is characterized as adult onset with progressive and permanent motor, mental, and cognitive symptoms that commonly result in death within 15 to 20 years of diagnosis [77]. The prevalence of HD was rated as too low in the past, before the identification of the underlying genetic mutation in 1993. As a result, the diagnosed prevalence of HD has been considerably higher in the post-diagnostic testing era [77]. HD is a member of the “proteopathies” group, which also includes Alzheimer’s disease, Parkinson’s disease, amyotrophic lateral sclerosis, and polyglutamine disorders [34]. The inheritance pattern is autosomal dominant and totally penetrating [76]. An expansion of the CAG (cytosine–adenine–guanine) trinucleotide sequence in the IT-15 (“interesting transcript 15”) gene encodes for huntingtin (HTT), which is a large protein of 3144 amino acids [8,44]. The IT-15 gene has a particular CAG sequence with up to 35 repetitions in a healthy person. However, individuals with more than 39 CAG repeats are more likely to develop the illness [8]. 

Mutant huntingtin causes neuronal malfunction and death at the cellular level through a variety of mechanisms, including the disruption of proteostasis, transcription, and mitochondrial function, as well as the direct toxicity of the mutant protein. Initial macroscopic modifications are observed in the striatum with the involvement of the cortex along with the progression of the HD. HD patients have atrophy of varying degrees in the white matter, thalamus, cerebellum, cerebral cortex, and various basal ganglia regions, in addition to striatal abnormalities. Structural/functional links among brain sections are gradually lost due to progressive brain atrophy [76]. Autonomic dysfunctions, cognitive/psychiatric symptoms, and abnormal involuntary movements are characterized by symptoms of adult-onset HD [8,44] Several effects, such as sleep disturbance, impaired glycemic control, and progressive weight loss could also be experienced [44]. The HD diagnosis criteria are positive genetic analysis or proven family history and the onset of motor impairment as determined by the Unified Huntington’s Disease Rating Scale-Total Motor Score (UHDRS-TMS). The score is between 0 (no motor abnormality suggestive of HD) and 4 (>99% based on HD). However, the pre-manifest stage of HD is defined as a course of 10–15 years from the onset of the disease that displays mild psychiatric, cognitive, and motor disruptions [8]. At present, HD is described as a cureless condition that worsens with time. The treatment consists of the pharmaceutical treatment of the alleviation of motor and mood-based symptoms. The FDA has only authorized two medications (tetrabenazine and deutetrabenazine) for HD-related movement disorders [76].

When combined with strategies that target glutamatergic neurotransmission, BDNF signaling, or mitochondrial function, medications that target mHtt DNA and RNA open up new, intriguing options and will likely produce superior results. Additionally, as HD has changed gut microbiome similar to other neurodegenerative diseases, addressing it may improve treatment outcomes. Genetic therapy applications are still in their infancy. There are still unanswered questions regarding non-selective mHtt lowering safety, the frequency of off-target effects in large populations, and the security of CRISPR therapies. Even though the growing number of persons participating in clinical trials demonstrates the scientific and medical community’s efforts to identify a disease-modifying drug or cure for this incapacitating neurological condition, a few recommendations are still required. In order to achieve results that are comparable across numerous sites and investigations, future clinical trials should incorporate techniques to reduce placebo and bias effects, employ standardized and validated rating scales, and conduct rigorous statistical analyses. The number of CAG repeats, for example, should be considered as they have a major impact on clinical trial outcomes. Finally, the development of more sophisticated assessments that are sensitive to the longitudinal cognitive changes in HD and the selection of appropriate cognitive endpoints will benefit the design of treatment studies seeking to halt the cognitive loss associated with this condition. Overall, considering these variables could aid in creating pre-clinical and clinical research designs that are more thorough [78,79].

## 3. Pharmaceutical Formulations with Proteins and Genes Targeting Neurodegenerative Disorders

Progressive neuronal malfunction and death are characteristics of neurodegenerative disorders (NDs). Neuronal vulnerability has been shown to be selectively affected by degradation, while the atrophy of damaged central or peripheral nervous system structures is a common symptom of NDs. Alzheimer’s disease is the most common form of such diseases, followed by Parkinson’s disease. Amyotrophic lateral sclerosis (ALS) and Huntington’s disease (HD) affect a smaller percentage of people, yet have far-reaching consequences [80]. Various small molecules, proteins and peptides [81], antibodies, as well as gene therapy drugs, can be applied for the management of genetic diseases, cancers, or neurodegenerative disorders. At present, gene therapy drugs mainly include plasmids, DNA, small interfering RNA (siRNA), microRNA (miRNA), and short hairpin RNA (shRNA) [82,83]. It is understandable that the aforementioned molecules are sensitive and easily degraded; thus, the development of efficient delivery vehicles that can protect them from degradation while effectively delivering them to brain or CNS tissues is critical. The most widely used carriers of genes are viral vectors [84], which have been gradually replaced by nanotechnology-based carriers. From the non-viral delivery vehicles, cationic polymers and lipid-based nanoparticles, such as cationic liposomes, are the most studied nanosystems, followed by inorganic nanocarriers, such as gold nanoparticles [85]. Two main ways have been identified for the delivery of the peptides/proteins or gene therapeutic molecules to the brain, which are either their encapsulation into the system or the conjugation or functionalization of them on the surface of the vehicles [86].

However, as it was already mentioned, drug delivery to the brain is challenging for the medical society due to the numerous barriers of the CNS and brain. The BBB is a major impediment to successful therapy because it blocks the transport of diagnostic and therapeutic chemicals while protecting brain tissue from hazardous compounds [87,88,89,90]. Moreover, the type of ND may be multisystemic, which can significantly complicate therapy. The death of certain kinds of neurons in NDs is not the product of a single pathogenic cause, but rather a series of detrimental molecular and cellular processes [91,92,93]. Nanopharmaceutical formulations could play a vital role in overcoming these obstacles.

**Figure 4:**
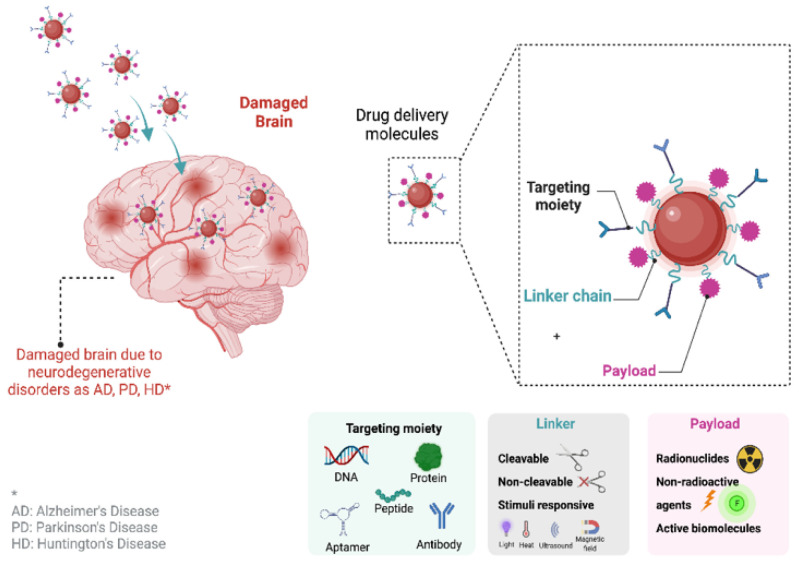
Various functionalized drug delivery systems have been employed to deliver active molecules to the brain, targeting neurodegenerative disorders. The modified nanoparticles carry targeting moieties, i.e., DNA, antibodies, peptides, aptamers, and proteins, and are able to encapsulate radionuclides, non-radioactive agents, as well as active molecules. In addition, the multifunctional carriers incorporate linkers so as to improve stability, higher payload, and stimuli responsive behavior (created with BioRender.com).

Nanotechnology-based materials have several benefits due to their size-related features, leading to considerable potential for medical and pharmaceutical applications, such as drug-targeted treatments, as well [53,94]. Moreover, the main problem with neurodegenerative disorders is that the novel drug candidates lack desirable pharmacokinetic and biopharmaceutical characteristics. Therefore, the design of suitable drug delivery systems is essential to ensure that only the targeted location receives the medication molecule. These new medication molecules are safer and more effective since they are based on nanotechnology. Additionally, nanomedicines have improved biopharmaceutical characteristics (adsorption, distribution, metabolism, and excretion) and minimize the dosage frequency. Moreover, the nanomedicines are able to deliver active molecules to specific cells, while they also provide sustained release patterns and an intracellular refuge against efflux or destruction [95,96,97]. Polymeric nanoparticles, lipid-based systems, liposomes, dendrimers, and magnetic nanoparticles are only some of the nanopharmaceutical formulations available for the treatment of NDs. Figure 4 summarizes the drug delivery systems that can penetrate the blood–brain barrier, targeting neurodegeneration.

It has already been mentioned that the therapies approved, to date, for neurodegenerative illnesses only address the symptoms. On the other hand, protein or gene-based treatments could positively impact the control of disease progression, according to the new data [98]. Therefore, the development of protein and gene-based targeted nanoformulations is important for the treatment of neurodegenerative diseases. In this review, the nanoformulations were categorized according to their used material.

### 3.1. Polymeric Nanoparticles

Polymeric nanoparticle-based therapies offer significant promise in the treatment of a wide range of disorders, due to their numerous properties, such as flexibility, easy modification, and promising architectural structures. Various advancements in polymerization methods, as well as the use of reactive, efficient, and orthogonal chemical modification reactions, have enabled the creation of multifunctional polymeric nanoparticles. These formulations allow for the assembly and transformation of nanoparticles with specific overall shapes and sizes, internal morphologies, external surface charges, and functionalization. Furthermore, through remote activation, the integration of particular capabilities can alter the reactivity of these nanostructures to certain inputs [99,100]. Table 1 summarizes the polymeric nanoparticles targeting neurodegenerative disorders.

At present, there has been a surge in interest in polymeric nanoparticles (NPs) because of their unique features that come with their minuscule size. It is possible to employ polymeric NPs for controlled release in order to protect drugs and other biologically active substances from exposure to the environment and increase their bioavailability and therapeutic index [121]. For example, Jeon et al. observed that poly(D,L-lactic acid-co-glycolic acid) (PLGA) nanoparticles containing the DBP (Vitamin-D-binding protein) were able to reduce Alzheimer’s disease-related pathology in 5XFAD mice. According to the results, the inhibition of amyloid beta peptides is shown to be successful when studied in vitro. Furthermore, in vivo experiments showed attenuated amyloid-beta buildup, neuroinflammation, neuronal death, and cognitive impairment [101]. 

**Figure 5:**
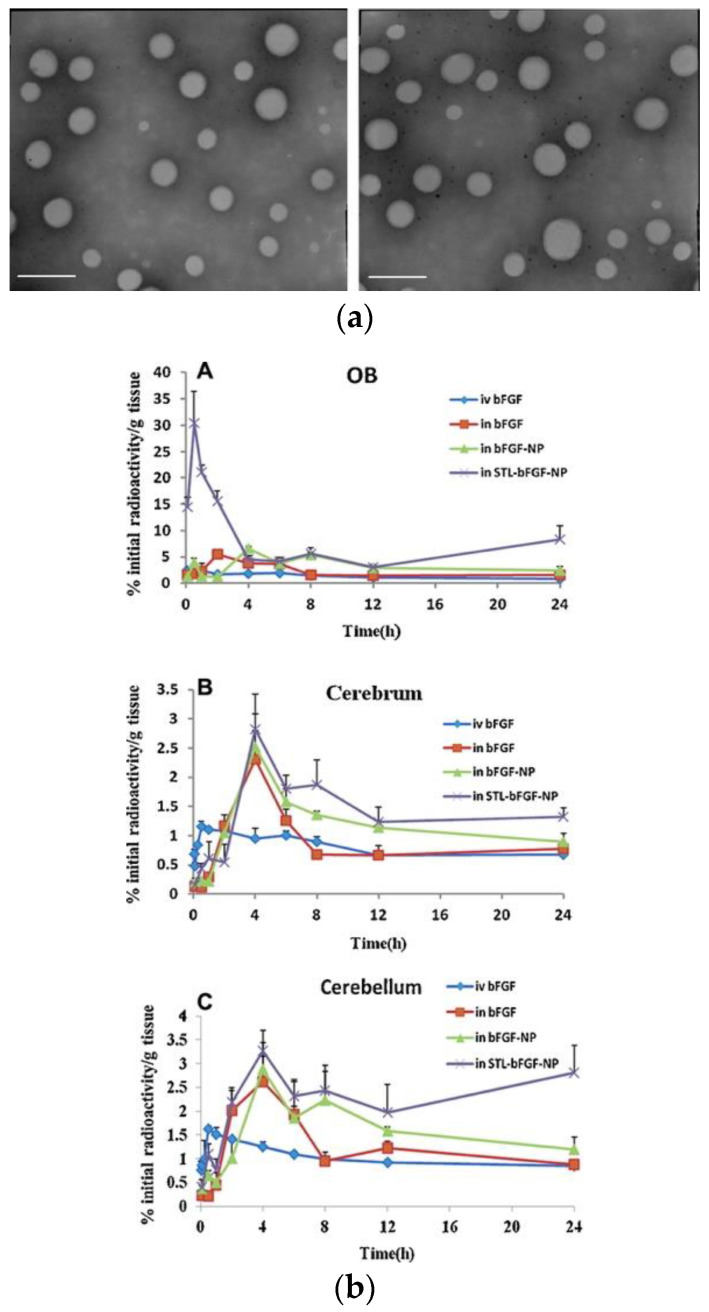
(**a**) The uniform shape of the bFGF-loaded lecithin PEG-PLGA nanoparticles; (**b**) brain distribution of the amount–time profiles of basic fibroblast growth factor in the olfactory bulb: OB (A), cerebrum: CR (B), and cerebellum: CL (C) following the intranasal administration of ^125^I-bFGF solution, ^125^I-bFGF-NP, and STL-^125^I-bFGF-NP, and an intravenous injection of ^125^I-bFGF solution. Reprinted from the International Journal of Pharmaceutics, 461, 11, Zhang et al. Intranasal nanoparticles of basic fibroblast growth factor for brain delivery to treat Alzheimer’s disease, copyright (2014), with permission from Elsevier [102].

There is great hope for the therapeutic molecules known as growth factors (GFs), which include bone morphogenetic proteins (BMPs), insulin-like growth factors (IGF-1/IGF-2), basic fibroblast growth factor (bFGF), and neurotrophins (nerve growth factor, NGF; glial-derived neurotrophic factor, GDNF; and brain-derived neurotrophic factor, BDNF). These GFs are generally located in the brain, where they play an important role during the many phases of development of the central nervous system (CNS) [122]. Zhang et al. administered bFGF-loaded lecitin-modified polyethylene glycol-polylactide-polyglycolide (PEG-PLGA) nanoparticles to male Sprague Dawley (SD) rats through an intranasal route for the management of Alzheimer’s disease. The nanoparticles were spherical, uniform, and exhibited a negative zeta potential, according to the results. Furthermore, the intranasal administration of the formulations resulted in 5.17 times larger areas under the concentration–time curve (Figure 5a,b) when compared with intravenous treatment. The neuroprotective effects of the formulations were also investigated, and the results reveal considerable improvements in spatial learning and memory [102]. 

**Figure 6:**
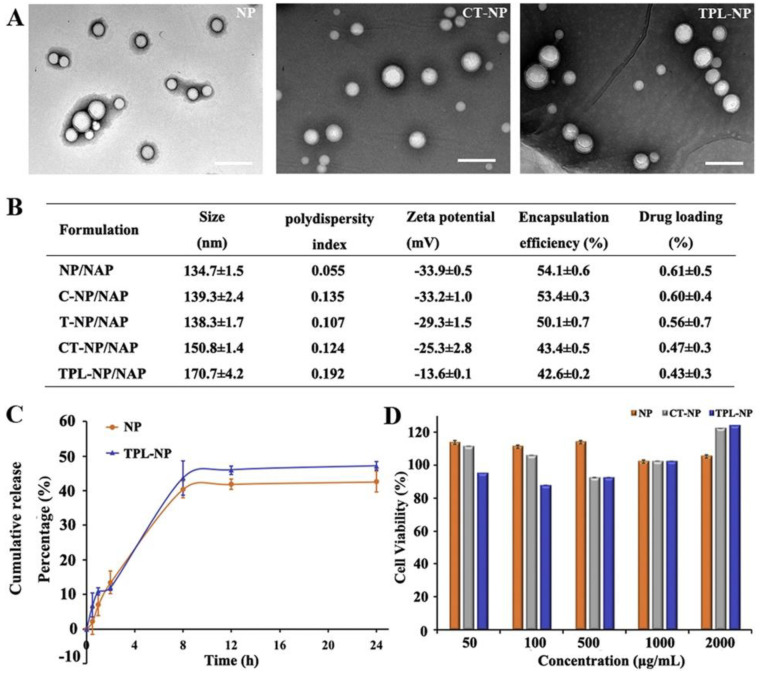
The characterization of PLA nanoparticles loaded with TPL peptides for AD management, developed by Guo et al. [110]. (**A**) TEM images of the developed TPL-modified pegylated PLA nanoparticles (**B**); various physicochemical properties of the prepared nanoparticles, such as size, polydispersity index, zeta potential, encapsulation efficiency, and drug loading (**C**); drug-release profiles of NAP peptide from NP/NAP or TPL-NP/NAP in PBS solution at pH 7.4 (**D**); HT22 cells’ viability of the developed nanoparticles. Reprinted from the Journal of Controlled Release, 320, 347–362, Guo et al. A dual-ligand fusion peptide improves the brain-neuron targeting of nanocarriers in Alzheimer’s disease mice, copyright (2021), with permission from Elsevier.

In another study, NGF-adsorbed poly (butly cyanoacrylate) (PBCA) nanoparticles used for the treatment of Parkinson’s disease were created. Polysorbate 80 was used to cover the nanoparticles that were prepared. It was revealed that amnesia was reversed, and memory and recognition were improved. Furthermore, the produced formulation reduced the primary symptoms of Parkinson’s disease, such as oligokinesia, rigidity, and tremors [103]. Guo et al. developed pegylated PLA nanoparticles modified with TPL as fusion peptides (Figure 6). The developed nanosystems showed improved BBB penetration and neuron-targeting efficacy, while the encapsulation of neuroprotective peptide NAP significantly improved reactive oxygen species scavenging ability and effectively protected microtubules from Aβ25-35-induced neurotoxicity [110]. Zhang et al. produced PEG-PLA nanoparticles and decorated them with TGN and QSH in order to improve their penetration into the BBB. According to the results, the peptide functionalization can improve the diagnosis and treatment of the nanoparticles [111]. Pegylated PLA nanoparticles were conjugated with Odorranalectin and loaded with the rocortin peptide as a possible therapy method for PD. The nanoparticles were evaluated for their efficacy on hemiparkinsonian rats indicating improved brain delivery and therapeutic efficacy for PD [112]. In another study, PEG-PLA nanoparticles were conjugated with TGN and QSH peptides and loaded with the H102 peptide as a dual system for AD treatment. The in vivo results reveal that the nanopaticles can possibly carry peptide or protein drugs, such as H102, and improve their penetration into the central nervous system, offering possible AD therapy [113].

It is rational to think that the use of PEG as part of the nanoparticles is very significant, and various studies involve its usage. Moreover, nanoparticles based on bis(pyrene)-Lys-Leu-Val-Phe-Phe-Gly-poly ethylene glycol (BP-KLVFFGPEG) were created to test their efficacy on Aβ amylods, which capture Aβ42 via the recognition and co-assembly of KLVFF. The co-assembly nanosystems would be a promising strategy for AD management [114].

**Figure 7:**
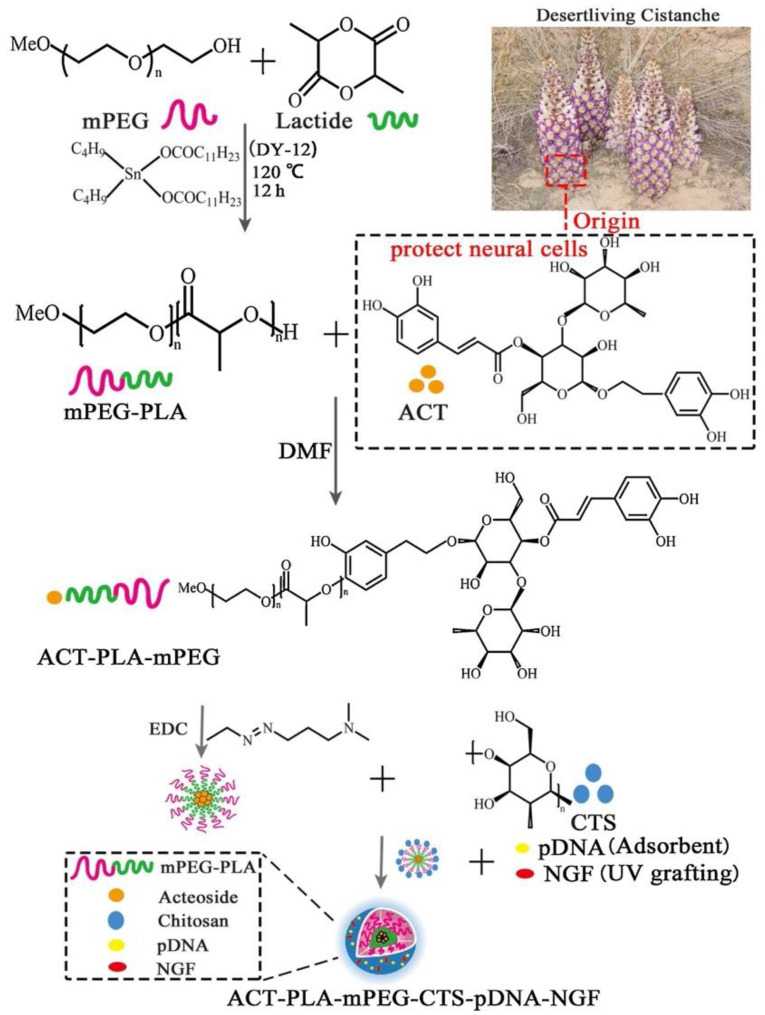
Schematic illustration of the prepared nanoparticles based on chitosan poly ethyleneglycol-poly lactic acid (PEG-PLA) nanoparticles coupled with NGF, acteoside, and pDNA. Reprinted from the Journal of Material Science and Technology, 43, Xue et al. Neuroprotective effect of chitosan nanoparticle gene-delivery system grafted with acteoside (ACT) in Parkinson’s disease models, 11, copyright (2020), with permission from Elsevier [106].

Gene therapy is another option for the treatment of ND diseases. Consequently, various genetic elements, such as small interfering RNA (siRNA), small hairpin RNA (shRNA), antisense oligonucleotides (ASOs), and cDNA, are being used, at present [9]. In one study, researchers developed novel polymeric nanoparticles incorporating Nogo receptor-siRNA and BDNF in order to understand their synergistic effect in terms of AD treatment. Therefore, crosslinked starch nanoparticles were developed by the reverse microemulsion technique, and then the prepared nanoparticles were modified by poly-lysine. Through this modification, the nanoparticles took full advantage of cationic properties. In vivo studies revealed that the BBB can be overcome by delivering biological drugs to the basal forebrain and hippocampus [104]. Moreover, Zheng et al. created a hybrid siRNA delivery technique to improve brain penetration and target amyloid plaques. To achieve the aforementioned goals, pEGylated Poly(2-(N,N-dimethylamino) ethyl methacrylate) (PEG-PDMAEMA) core materials were combined with two d-peptides, a CGN for brain penetration and a QSH for β-amyloid binding. The results demonstrate that the formulations are lower in cytotoxicity and siRNA is protected against enzyme degradation. The data also reveal a 18.5 percent decrease in protein levels [105]. In another study, researchers studied GDNF gene therapy for the treatment of Parkinson’s disease. pDNA-encoding human GDNF nanoparticles were prepared and administered intranasally to transgenic rats. The prepared nanoparticles showed greater neuroprotection compared to naked pDNA [123]. For the treatment of PD, Xue et al. produced chitosan poly ethyleneglycol-poly lactic acid (PEG-PLA) nanoparticles coupled with NGF, acteoside, and pDNA (Figure 7). The created nanoparticles had a strong in vitro neuroprotective impact, while the behavioral problems of sick mice were improved in vivo [106]. Liu et al. developed a core–shell hybrid system called rabies virus glycoprotein (RVG) peptide-modified exosome curcumin/phenobornic acid-poly(2-(dimethylmino)ethyl acrylate) nanoparticle/siRNA targeting α-synuclein aggregates. The prepared nanoparticles were designed as nanoscavengers for clearing α-synuclein aggregates and reducing their cytotoxicity in Parkinson’s disease neurons. the in vivo studies presented improved motor behavior results. In addition, it was also observed that the prepared formulations were good nanoscavengers for cleaning immune activation [107]. In another study, Gan et al. developed miRNA-124-loaded RVG29 surface-modified polymeric nanoparticles for the treatment of PD. The mRNA levels of induceable nitric oxide synthase (iNOS), tumor necrosis factor-alpha (TNF-α), and interleukin-6 (IL-6) were dramatically increased because of the study’s results. Furthermore, the proinflammatory factors were decreased and neuroprotective compounds were elevated following transfection with the produced formulations. Consequently, the researchers hypothesized that these nanoparticles may decrease pro-inflammatory signals, while also enhancing neuroprotection properties [108]. Lv et al. created pegylated PLGA nanoparticles in another investigation for AD. Epigallocatechin-3-gallate (EGCG) and β-site amyloid precursor protein (APP)-cleaving enzyme 1 antisense (BACE1-AS) shRNA-encoded plasmid were introduced into the nanoparticles. The RVG29 peptide was used to multifunctionalize and target the created nanoparticles. Flow cytometric study, biocompatibility testing, pharmacokinetic analysis, Western blot analysis, and the Morris water-maze test were used to evaluate the formulations. According to the results, the co-delivery of a therapeutic gene (shRNA) and EGCG in a multifunctional nanocarrier might result in higher therapeutic concentrations in the brain, making it a promising technique for treating Alzheimer’s disease [109]. 

According to the study conducted by Liu et al., RVG29 was applied as the brain-targeted modification for PEGylated dendrigraft poly-l-lysines for the systemic administration of plasmid DNA-encoding BACE1-AS shRNA. The system presented a reduction in the key enzymes responsible for amyloid-β formation and neurofibrillary tangles. Furthermore, the memory loss of AD mice was also improved [115]. Saraiva et al. developed PLGA nanoparticles loaded with miRNA-124 for the treatment of Parkinson’s disease. Nanoparticles containing perfluoro-1,5-crown ether (PFCE) and coated with protamine sulfate were prepared and used to bind miRNA-124. The capacity of NPs to transmit miRNA-124, neurogenesis, and brain repair was tested by the researchers. The study’s results reveal that the created formulations offer convincing evidence to support the use of miRNA-124 NPs as a potential therapeutic method for boosting endogenous brain-repair mechanisms in neurodegenerative conditions [116]. Valenza et al. produced cholesterol-loaded PLGA NPs in order to study their delivery to the brain and restore abnormal cholesterol homeostasis. The decorated nanoparticles with g7 glycopeptide showed an improvement in BBB targeting and brain accumulation. Finally, the nanoparticles were studied in an HD model so as to examine if they could improve synaptic and cognitive dysfunctions in R6/2 transgenic mice. The results are promising and the system can be applied for HD management [117].

In another study, Helmschrodt et al. developd polyethyleneimine nanoparticles loaded with siRNA to reduce α-synuclein expression for the treatment of PD. They administered the prepared formulation by intracerebroventricular infusion into the PD modeling mice. The prepared formulation was distributed across the CNS down to the lumbar spinal cord following a single-dose administration. The mRNA expression of SNCA in the striatum was reduced by 65% and SNCA protein was decreased by 50%. Moreover, mice did not present any kind of toxicity or adverse effects [118]. For the treatment of ND, Sanches-Ramos developed chitosan-mangafodipir nanoparticles for the intranasal transport of siRNA and DNA to the brain. Manganese nanoparticles containing anti-eGFP siRNA were investigated in cell cultures of the eGFP-expressing cell line of murine fibroblasts for this reason (NIH3T3). The best nanoparticles were then tested in mice in vivo. The nanoparticles were categorized as efficient in lowering GFP mRNA expression in the olfactory bulb, striatum, hippocampus, and brain of Tg GFP+ mice. The RFP was expressed in various brain regions following an intranasal instillation of mNPS loaded with dsDNA-encoding RFP [119]. Finally, chitosan nanoparticles loaded with anti-HTT siRNA were studied in vivo in a transgenic YAC128 mouse model of HD. According to the results, the chitosan nanoparticles, when intranasally administered, decrease HTT mRNA expression and therefore can act as possible therapeutic startegies for HD [120].

### 3.2. Lipid-Based Nanoparticles

Lipid-based nanocarriers, such as liposomes, solid lipid nanoparticles, and nanostructured lipid carriers, are applied for various disorders due to their unique characteristics. Therefore, various lipid nanocarriers can be found to be loaded with genes or proteins for AD or PD. Table 2 summarizes the lipid-based and inorganic carriers for neurodegerative disorders.

Liposomes have been used for a variety of purposes, since they are able to originally self-assemble into vesicular structures that have been continually broadened and enhanced. Based on the lipid content, these configurations exist in a variety of forms and sizes. Liposomes are frequently utilized to carry therapeutic molecular payload, such as DNA [124]. It has been suggested that the brain-derived neurotrophic factor (BDNF) is actively developed and used in cortical circuits during life in order to sustain neuronal function and synaptic plasticity. Therefore, mannose functionalized liposomes, along with cell-penetrating peptides, were used as the vehicle for the delivery of BDNF against AD. The developed lipid-based nanoparticles exhibited improved BDNF expression, while they reduced the toxic Aβ peptides when applied to APP/PS1 mice brains. Another significant outcome was that the bifunctionalized nanoparticles did not present any adverse effect, revealing their potent application as a safe and effective strategy for AD [125]. 

**Table 2:** Pharmaceutical formulations with proteins and genes targeting neurodegenerative disorders using lipid-based and inorganic nanoparticles.

Active Molecule	Formulation	Targeting Disease	Method	Ref.
BDNF	Mannose functionalized liposomes	AD	In vivo	[125]
Vgf (non-acronymic), a neurotrophin-stimulated protein	Liposomes	AD	In vivo C57BL/6J mice	[126]
Retro-inverso peptide RI-OR2-TAT	Liposomes	AD	In vitro	[127]
GDNF plasmid-	Pegylated liposomes	PD	In vivo 6-hydroxydopamine (6-OHDA) mice	[128]
NGF gene	1,2-distearoyl-sn-glycero-3-phosphoethanolamine–poly(ethylene glycol) phospholipid-based liposomes	PD	In vivo C57BL/6J mice	[129]
GDNF	Nanostructured lipid carriers (NLCs)	PD	In vivo MPTP mice	[130]
Conjugated transferrin	Solid lipid nanoparticles (SLNs)	AD	In vitro	[131]
Erythropoietin	Solid lipid nanoparticles (SLNs)	AD	In vivo Albino male Wistar rats	[132]
GDNF	Chitosan-coated NLC	PD	In vivo rats	[133]
BACE1 siRNA	Chitosan-coated SLNs	AD	In vitro	[134]
siRNA	RGV-anionic liposomes	PD	In vitro	[135]

The same research group developed liposomes for the delivery of Vgf, which is a neurotrophin-stimulated protein found in low levels in AD brains. The liposomal formulations were functionazlied by mannose and brain-targeted cell-penetrating peptides (chimeric rabies virus glycoprotein fragment, rabies virus-derived peptide, penetratin peptide, or CGNHPHLAKYNGT peptide). It was revealed that lipid nanoparticles presented in vitro and in vivo biocmpatibility while they were able to penetrate the BBB. Consequently, the nanoparticles can be characterized as an efficacious and safe gene delivery system for the treatment of AD [126]. Various studies examined the use of liposomes for targeting Aβ toxicity. More specifically, liposomes functionalized with retro-inverso peptide RI-OR2-TAT (Ac-rGffvlkGrrrrqrrkkrGy-NH2) presented a reduction in the aggregation and toxicity of Aβ. Although the study lacks an in vivo analysis, the biodistribution of healthy mice evidences the penetration of liposomes into the brain. Moreover, the peptide-inhibitor nanoparticles could modify AD treatment [127]. Microbubbles containing GDNF plasmid-loaded pegylated liposomes were created by Yue et al. for the treatment of Parkinson’s disease. The authors examined GDNF plasmid gene transfer using PEGylated liposome-coupled microbubbles (PLs-GDNF-MBs) for behavioral impairment and neuron death in a PD rat model. The delivery could be activated by ultrasound. The results show that delivering PLs-GDNF-MBs into the brains of the rats with Parkinson’s disease using an MRI-guided focused ultrasound helps to ameliorate behavioral impairments and neuron loss [128]. Using a 1, 2-distearoyl-sn-glycero-3-phosphoethanolamine–poly(ethylene glycol) phospholipid, dos Santos Rodrigues et al. developed brain-targeted gene delivery systems with prolonged systemic circulation and enhanced cellular penetration. The modification was performed by coupling the transferrin (Tf) ligand and penetratin (Pen) peptide to liposomes. The NGF gene was then added to the manufactured liposomes. This resulted in Tf and Pen-modified liposomal nanoparticles encapsulating plasmid NGF traversing the BBB model and transfecting primary neuronal cells. The treatment of primary neuronal cells with liposomes boosted the NGF expression, which resulted in an increase in the presynaptic marker synaptophysin. The PenTf-liposomes carrying pNGF were administered to APP/PS1 mice (3 months old) for 4 weeks (1 injection per week) to reduce hazardous soluble and insoluble Aβ-peptide levels (p < 0.05) compared to those levels in untreated APP/PS1 animals [129]. The alpha-synuclein (α-syn) deposition in Lewy bodies (LBs) is one of the main neuropathological signs of PD. Consequently, novel liposomes able to deliver small interfering ribonucleic acid (siRNA) for the reduction in neuronal α-syn by RNA interference were prepared by Schlich et al. [135]. The RVG-decorated anionic liposomes were observed to induce α-syn gene silencing in primary neurons without altering the cell viability [135].

Solid-lipid nanoparticles and nanostructured lipid carriers belong to an interesting category of lipid-based nanocarriers targeting neurodegenerative disorders. Kuo et al. produced multiple-component solid-lipid nanoparticles containing conjugated transferrin. The SLNs were supplemented with rosmarinic acid, curcumin, and quercetin for an increased neuronal apoptosis-fighting action. It was determined through testing that the generated formulations were promising for the treatment of AD [131]. Erythropoietin (EPO), a hematopoietic factor, has been suggested as a potent neuroprotective candidate for neurodegenerative disorders. EPO-loaded SLNs were studied for their effects on the beta-amyloid, and characterized as a safe and efficient system for AD management. It was revealed that the memory of the animals was significantly restored in cognitive-deficit rats. Moreover, the nanosystems reduced oxidative stress, the ratio of ADP to ATP, and the buildup of beta-amyloid plaques in the hippocampus [132]. 

A nanoformulation created by Hernando et al. was applied to enhance brain targeting in PD via intranasal administration. As a result, GDNF was encapsulated in nanostructured lipid carriers (NLCs). The surface of these carriers was changed with the transactivator of the transcription peptide (TAT). An 1-methyl-4-phenyl-1,2,3,6-tetrahydropyridine (MPTP) mouse model was used for the in vivo investigation after nanoparticles were characterized in terms of their physical and chemical properties. Motor recovery was validated by immunohistochemical investigations, which presented the highest numbers of tyrosine hydroxylase (TH+) fibers and TH+ neurons in the substantia nigra in the CS-NLC-TAT-GDNF-treated group [130]. Chitosan-coated NLCs loaded with GDNF were developed by Gartziandia et al. for the treatment of Parkinson’s disease. The new formulation was intranasally delivered and tested on 6-OHDA rat models. A possible promising treatment plan for Parkinson’s disease could be achieved by these formulations with an excellent encapsulation efficiency [133]. For the treatment of Alzheimer’s disease, Rassu et al. created solid-lipid nanoparticles loaded with BACE1 siRNA. The RVG-29 was used to increase the cell penetration of the nanoformulations that was prepared. Chitosan was also used to coat the formulations. Chitosan coating resulted in protected siRNA and enhanced muco-adhesion, as well as improved the duration of time spent in the nasal cavity. Finally, chitosan coating enhanced siRNA absorption into Caco-2 cells further [134]. 

### 3.3. Other Nanosystems 

Other nanocarriers for the targeting of neurodegenerative disorders include inorganic nanocarriers as well as densritic systems. Table 3 presents a summary of the drug formulations that use proteins and genes to treat neurodegenerative diseases.

Zilony-Hanin et al. [136] prepared NGF-loaded porous silicon nanostructures for the treatment of AD. This aimed to release the NGF for more than 30 days. These formulations were administered by the implantation of silicon nanoparticles containing chips over the dura mater and via silicon microparticle biolistic bombardment by an opening in the skull developed by a pneumatic gene gun. This study showed that the degradable silicon microcarriers can act as a potent system for the localized delivery of NGF to the brain [136]. Zhang et al. [145] developed a multi-strategy peptide (MOP), an ingenious apolipoprotein E mimetic peptide, which was able to be self-assembled into nanorods and was capable of reducing Aβ deposition via inhibiting Aβ aggregation and, at the same time, accelerating Aβ clearance. The nanorods could permeate the blood–brain barrier (BBB) to address the poor delivery to the brain issues, which could be used as a management strategy for AD [145].

Magnetic nanoparticles loaded with a plasmid containing a α-synuclein RNAi were developed by Niu et al. [137] for the treatment of PD. The iron oxide nanoparticles were coated with oleic acid. An N-isopropylacrylamide derivative was photo-immobilized on oleic acid molecules, while shRNA was absorbed. For NGF absorption, they employed the same technique. The multifunctional superparamagnetic nanoparticles with shRNA for α-syn provided efficient repair in a PD model, as evidenced by the data [137]. 

Gold nanoparticles have been widely used for the diagnosis and therapy of neurodegenerative and other disorders. Therefore, Zhang et al. examined the neuroprotective effects of maize tetrapeptide-anchored gold nanoparticles against l-glutamic acid-induced PC12 cell apoptosis and a murine Alzheimer’s disease model induced by aluminum chloride and d-galactose. The authors concluded that the gold nanoparticles may have an impact the AD treatment [140]. An interesting study conducted by Prades et al. demonstrated that gold nanoparticles conjugated to the peptide CLPFFD could diminish the toxic aggregates of β-amyloid; therefore, a gold nanoparticle–CLPFFD conjugate loaded with the peptide sequence THRPPMWSPVWP was developed and studied in vitro and in vivo. It was revealed that this sequence interacted with the transferrin receptor present in the microvascular endothelial cells of the BBB, and could improve the permeation process, which is essential for AD management [139]. Liu et al. [138] prepared gold nanoparticles for the therapy of PD. Gold nanoparticles were loaded with pDNA to inhibit α -synuclein in this investigation. The results show that NPs present strong neuroprotective benefits in PD mice, not only in motor but also in non-motor dysfunctions, suggesting a potential gene therapy technique for PD [138]. In another study, gold nanorods stabilized with polyethylene glycols and modified with the D1 peptide that recognizes toxic aggregates of Aβ, a peptide involved in Alzheimer’s disease (AD) and Angiopep 2 that can be used to deliver nanorods to the mammalian central nervous system, were developed by a research group. The nanorods were able to penetrate the neuron cells and reduce the Aβ peptide leading to interesting results for AD management [142]. Hu et al. [141] developed gold nanoparticles for neuroprotective effects in PD models. The fold nanoparticles loaded with plasmid DNA targeted PC12 cells. The nanosystems inhibited the apoptosis of the aforementioned cells and dopaminergic neurons. Finally, the gold nanoparticles were able to penetrate the BBB in vivo, and therefore have great therapeutic efficacy for PD management [141].

Kim et al. created thermoresponsive and biodegradable linear–dendritic nanoparticles using poly(N-isopropylacrylamide), poly(L-lactic acid), and poly(L-lysine) dendrons. The aim of the study was to examine the long-term release of NGF in response to temperature changes. The study’s results reveal a novel idea for treating Alzheimer’s disease and other neurological illnesses by employing thermoresponsive and biodegradable linear–dendritic nanoparticles for the thermally targeted and sustained release of NGF and other protein therapeutics [143].

Cyclodextrins are sugar molecules bound together in rings of various sizes. They can host various molecules, and they are able to improve solubility and drug delivery properties. For example, chemically modified CD-based nanoparticles loaded with siRNA were synthesized in order to target the huntingtin (HTT) gene. The system was functionalized with the rabies virus glycoprotein (RVG) which, as already stated, can improve BBB permeation. The in vitro results of a BBB model of HD incorporating the human cerebral microvascular endothelial cell line (hCMEC/D3) in combination with the rat striatal embryonic neuronal cell line ST14A demonstrate that the cyclodextrin nanoparticles can penetrate the brain endothelial cells, release the encapsulated siRNAs into the cytoplasm of neuronal cells, and mediate the downregulation of HTT [144].

## 4. The Use of Radiopharmaceuticals for the Diagnosis and Therapy of Neurodegenerative Diseases

It has been already mentioned that the understanding of the pathophysiological mechanism of neurodegeneration is critical in order to develop efficient therapeutic strategies. Therefore, imaging methodologies, such as magnetic resonance imaging (MRI), positron-emission tomography (PET) [146], and single-photon-emission computed tomography (SPECT), can be used for the identification of favorable diagnostic biomarkers, as well as potential treatment targets for neurodegenerative diseases. Brain and CNS imaging via nuclear medicine provides hopeful results [147].

**Figure 8:**
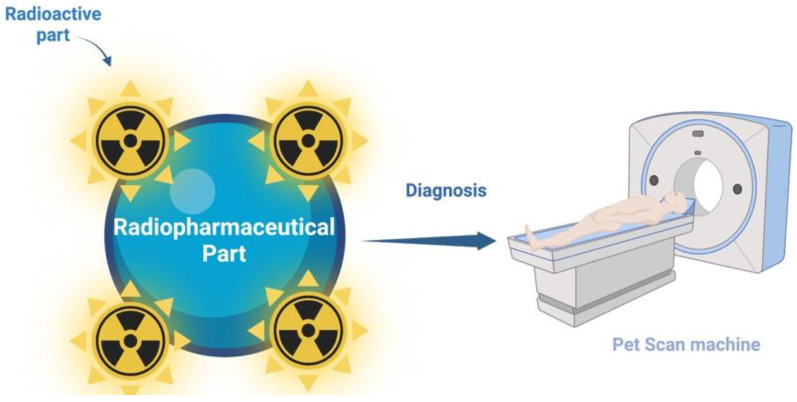
The scheme of radiopharmaceuticals. Radiopharmaceuticals contain radioactive and pharmaceutical parts. They can be applied for diagnosis purposes, especially PET-scan techniques as well as others (created with BioRender.com).

Radiopharmaceuticals consist of radionuclides and pharmaceutical parts. The radionuclides emit different rays and they bind to pharmaceutical parts so as to obtain ideal radiopharmaceuticals for the targeting of specific organs, tissues, or cells in the human body (Figure 8). They are mostly used for the diagnosis and treatment of diseases. For diagnosis, the radiopharmaceuticals are composed of radionuclides that contain gamma rays, while the pharmaceutical part encapsulates a lower quantity of drugs than the therapeutic-effect dose [148]. The therapeutic radiopharmaceuticals are composed of therapeutic radionuclides that emit alpha and beta particles and their therapeutic parts. The radionuclide parts are combined with the pharmaceutical parts of high affinity to a tissue or a cell using a linker molecule. In some cases, therapeutic radiopharmaceuticals only have a radionuclidic part, if it has an available biological feature. It is believed that the clinical administration of diagnostic radiopharmaceuticals is greater than therapeutic radiopharmaceuticals [149]. Scintigraphy is an important process that uses diagnostic radiopharmaceuticals to image organs or tissues of interest [150]. Gamma cameras, single-photon positron emission tomography (SPECT), and positron emission tomography (PET) are medical devices that can detect radioactive rays emitted by radiopharmaceuticals [151]. Furthermore, the administration of radiopharmaceuticals with medical imaging devices generated by non-invasive approaches reflects the function of the organ or tissue under investigation. It is estimated that the clinical usage of therapeutic and diagnostic radiopharmaceuticals will rapidly increase. As a result, detailed information on the preparation of radiopharmaceuticals, quality control, and specifications is required to provide medical authorities with better access to information on the characteristics, treatment, and images of various types of tumors.

**Figure 9:**
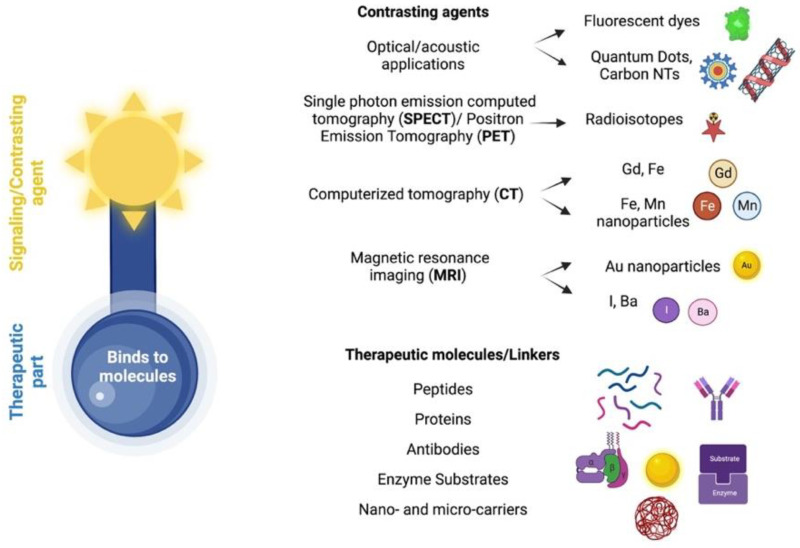
The general structure of theranostic radiopharmaceuticals. Their therapeutic part binds to the molecule while the signaling part or contrasting agents are responsible for their diagnostic ability. Contrasting agents include fluorescent dyes, quantum dots, carbon NTs, radioisotopes, and inorganic particles based on Au, Fe, Mn, Gd, I, and Ba. Therapeutic molecules can be peptides, proteins, antibodies, and enzymes (created with BioRender.com).

Theranostic radiopharmaceuticals can diagnose and treat diseases at the cellular and molecular levels [152]; however, new theranostic agents are needed, and many are under development, at present [153]. In fact, the efficiency of disease detection is problematic because a sufficient number of cells does not exist for a successful diagnosis. Ideally, while the detection limit of unhealthy cell numbers can be 109 per 1 g, patients with 1012 unhealthy cell numbers are believed to be in the last stage of disease. Figure 9 depicts the general structure of radiopharmaceuticals and their theranostic uses; the therapeutic molecules of theranostic radiopharmaceuticals involve chemotherapeutic molecules, proteins, peptides, genes, as well as other genetic molecules. On the other hand, diagnostic molecules found in theranostic radiopharmaceuticals include quantum dots [154], superparamagnetic iron oxides, radionuclides, as well as heavy elements, i.e., iodine [155] for magnetic resonance imaging (MRI), nuclear imaging, and computed tomography.

Although radiopharmaceuticals are promising imaging modalities for neurodegenerative disorders, their clinical use is impacted by properties, such as pharmacokinetics, pharmacodynamics, diagnostic/therapeutic efficacy, adverse reactions, and dosage of radiopharmaceuticals. These parameters affect the in vivo stability of any radionuclide/carrier complex and determine their safety in use as diagnostic or therapeutic objectives. The radioactivity of radiopharmaceuticals also interacts with the pharmacokinetic data. Prior to any human trial, animal studies should be performed if relevant or superseded by the data obtained from patients. There are numerous general preclinical and clinical questions that can be addressed using radiopharmaceuticals in the development of pharmaceutical products. The choice of reagents for radiopharmaceuticals, whether to use a therapeutic antibody or develop another format (antibody fragment or small molecule), needs to be considered according to the questions concerning the radiopharmaceutical applications (Table 4).

Medical Internal Radiation Dosimetry (MIRD) can calculate the radiopharmaceuticals and their absorbed dose in organs. The cumulative radioactivity in a desired cell/tissue/organ is very significant and is therefore calculated according to the origin of the data used, such as animal studies or measurements in humans. Physical parameters, such as absorbed dose by target organs per unit of cumulated activity in desired cell/tissue/organ, should also be acquired from MIRD schedules. The International Commission on Radiological Protection (ICRP) presents the effective dose-equivalent value by using the current weighting factors. These weighting factors are not suitable for children, pregnant women, or elderly patients. The radioactivity of radiopharmaceuticals should be rearranged for use in such patients, and modifications should be made in terms of radioactivity. The unit must be milligrays per unit of activity administered: mGy/MBq. The estimation of the radiation dose must be summarized in terms of the effective dose equivalent using the weighting factors presented by ICRP. The unit must be millisieverts per unit of activity: mSv/MBq [151,156].

The present research focuses on membrane receptors, kinase-linked receptors, nuclear receptors, enzymes, genes, and transporters to achieve successful delivery in neurodegenerative disease. The expression of genes encoding both in vivo and in vitro enzymes, transporters, or receptors has often been studied to prove their pharmacological activities. Based on the peptides and genes for targeting neurodegenerative disorders, these systems are combined with radionuclides, different dyes, optical markers, and MRI markers. The mechanism is based on binding, activating, or entrapping imaging markers, which can play the role of surrogate markers of gene expression at the desired site. There are several imaging modalities used to image one or multiple reporters, i.e., optical imaging (fluorescence as well as luminescence), MRI, and SPECT or PET radiopharmaceutical imaging. 

Among these imaging systems, the radionuclide imaging method has been characterized as sensitive and reproducible, as well as a non-invasive method for the characterization of neurodegenerative disorders. Furthermore, these systems provide sufficient image details and structures that facilitates the clinical translation of a cell, tissue, and organ. Because of their high sensitivity and low cost, SPECT, PET, and MRI are some tools used for the molecular imaging of metabolic alterations in neurodegenerative disorders. These imaging methods can determine changes in neurons, hemispheric asymmetry, changes in glucose levels, and other indicators of the differences between different types of dementia [157,158,159,160,161]. 

Somatostatin receptors (SSTRs) are mainly used as G-protein-coupled receptors. They reduce cell proliferation and increase apoptosis by changing the levels of hormones and proteins necessary for cell growth and proliferation. Thus, they prevent the uncontrolled proliferation of cells. They have five receptor subtypes, namely, somatostatin receptor-types 1–5 (SSTR1-5). SSTR2 has an important role in differentiated neuroendocrine carcinomas. It affects both normal and tumor tissues at very low levels. Additionally, their inhibitory effects have been observed on cell proliferation and the endocrine release of active hormones. There are some examples of clinically available octreotide-based PET radiopharmaceuticals, which can translate the use of the human SSTR2 gene (hSSTR2) as a reporter system to quantitate gene delivery. In a study, SSTR2 radiolabeled with Yitrium-90-DOTATOC (90Y-DOTATOC) and Lutetium-177- DOTATATE (177Lu-DOTATATE) was used on neuroendocrine tumors (NETs) as a new radiopharmaceutical therapy. They presented high-absorbed doses in tumors expressing SSTR2 and the possibility for desired delivery. Furthermore, a high survival rate in patients was reported clinically. When the radiolabeled complexes called 90Y-DOTATOC-SSTR2 and 177Lu-DOTATATE-SSTR2 were compared to 177Lu-DOTATATE, the survival rate of patients was observed to be higher than in patients treated with 177Lu-DOTATATE. In addition, while 90Y-DOTATOC-SSTR2 is found to be more effective for larger tumors, 177Lu-DOTATATE-SSTR2 is more effective for smaller tumors. Except for these examples, the administration of 99mTc-hydrazinopyridine-3-carboxylic acid (HYNIC)-SSTR2 and 68Ga- and 18F-labeled SSTR2 is increasing for tumor detection in patients with NETs because of the higher sensitivity of this technique and the shorter time needed for the investigation in comparison to SPECT imaging [162,163].

It has been suggested that MRI is one of the the most commonly used imaging techniques for brain and nerve cell diseases. It has high-spatial and -contrast-resolution capabilities that can assess the structural brain alterations occurring during neurodegeneration associated with aging and disorder processes. A recent study focused on diffusion kurtosis imaging to distinguish gray-matter aging patterns and exhibit salient macro–microstructure relationships in aging. The diagnosis of AD can be performed according to the MRI system in terms of the value of multiple, visual, rating scales. Zhao et al. [164] used a PET imaging system with a new diagnostic radiopharmaceutical to present a quantitative analysis of tau accumulation in healthy, older adults, mild cognitive impairment, and AD patients [164]. In another study, ^18^F-AV1451 was used to identify regional brain tau-protein accumulation in participants of different cognitive statuses and observe the correlations between cerebrospinal fluid (CSF) biomarkers or the Mini-Mental State Examination (MMSE), according to the 18F-AV1451 standardized brain-uptake-value ratio (SUVR). The distribution of ^18^F-AV1451 in Ʈ protein in AD patients was shown via the PET system. Similar results were obtained via 18F-FDG PET and MRI imaging, while revealing differences in the underlying physiological importance. The brain-activity mechanism was also identified via the PET-MRI hybrid-imaging modality. Dopamin analogs were radiolabeled with various radionuclides to aid in the early diagnosis of AD and estimation of disease severity. Levodopa is a dopamine analog with unfavorable effects on dopamine-replacement active molecules that can alleviate akinesia found in AD patients. The ^123^I- N-&-fluoropropyl-2b-carbomethoxy-3b-(4-iodophenyl) nortropane (I-123 FP-CIT) was developed as a SPECT radiopharmaceutical to evaluate the alterations in dopaminergic innervation that occur when AD is progressing. In addition, critical alterations of the nigrostriatal dopaminergic innervation were observed in AD patients [165,166,167]. AD and Parkinson’s disease (PD) are the most commonly significant neurodegenerative disorders and are related to population aging and increase proportionally with age. The aggregation of Ʈ proteins and misfolding are common in these defects. The differential diagnosis of AD is determined by the presence of a hyperphosphorylated Ʈ protein and amyloid peptide (Aβ). In PD, the formation of insoluble fibrils, which are the primary structural components of Lewy bodies (LBs) and neurites (LNs), is characteristic of the diagnosis of the disease. Some radiopharmaceutical examples for both disorders are presented in Table 5.

To date, the classification of neurodegenerative diseases is constantly changing and progressing. Predominantly, phenotypical definitions are being increasingly substituted, at least in the research settings, by classifications that include biomarkers for the underlying pathophysiological processes, and thus lead to a more biological definition of neurodegenerative diseases [184]. This development is of great significance for nuclear medicine, as molecular imaging using PET tracers may provide biomarker information, e.g., [18F]FDG as a biomarker of neuronal injury [185]. 

## 5. Conclusions and Future Perspectives

Neurodegenerative disorders are devastating diseases that affect millions of people worldwide. The use of nanoformulations for the management of neurodegenerative diseases, such as Alzheimer’s, Parkinson’s, and Huntington’s, seems to be promising; however, which pharmaceutical molecule can effectively target these diseases is a crucial question to answer. 

Gene and protein therapeutic strategies are an emerging field for the targeting of neurodegenerative diseases; gene therapy can deliver proteins and key molecules into the brains’ of people suffering from such diseases.

Although there is not enough clinical trial data to support the use of gene and protein delivery for the management of NDs, it can be concluded that the future of medicine belongs to such molecules because conventional therapies for neurodegenerative diseases do not produce any significant results. The main drawbacks of such molecules are their half-life, degradation, and possible adverse effects.

Nanotechnology-based carriers have been widely used as vehicles for the delivery of sensitive molecules, since they can protect them from degradation. A very important category of nanoparticles are those based on polymers. Polymeric nanoparticles can be easily functionalized by peptides and easily penetrate the brain barriers. In addition, polymeric nanocarriers can encapsulate genes or proteins and protect them while targeting specific brain regions or cells. According to the literature, poly(ethylene glycol), poly(lactic acid), and poly(lactic-co-glycolic acid) have been widely explored as components of these nanosystems; this is attributed to their favorable hydrophilicity, which improves brain penetration. Nonetheless, there are numerous polymers that should be studied as potent vehicles for biological molecules against neurodegenerative disorders. Natural polymers, such as chitosan or alginic acid; synthetic polymers, such as poly(ε-caprolactone); and derivatives of them can be used as novel nanovehicles of peptides and genes. Moreover, such polymers can also be surface functionalized with the delivery molecule or encapsulate it.

Other nanocarriers of biological molecules include lipid-based products, such as liposomes or solid-lipid nanoparticles and nanostructured lipid carriers. The lipid-based nanovehicles are frequently used in the nanomedicine field, especially for the delivery of sensitive molecules to the brain. In fact, such lipid molecules have been able to penetrate the brain barriers and effectively administer drugs to the tissues. Nonetheless, none of these formulations have been granted market approval, and major steps should still be taken to ensure their use in the pharmaceutical field. Moreover, in the last decade, inorganic carriers, such as gold or silicon-based nanoparticles, have attracted the interest of researchers due to their versatile properties as both imaging and therapeutic vehicles. As we mentioned for lipid-based nanocarriers, inorganic nanocarriers have also not been approved for use in the market as either diagnostic or therapeutic modalities. There should be a rapid increase in clinical trials in order to observe any inorganic nanomaterials in the market. Consequently, there is still a long road to the approval of inorganic nanosystems for the management of neurodegenerative diseases. 

An interesting fact is the use of radiopharmaceuticals for protein and peptide delivery to the brain and for the simultaneous diagnosis of neurodegenerative diseases. Various radiopharmaceuticals have been used for Alzheimer’s and Parkinson’s diseases; however, there are not any sufficient applications for Huntington’s disease. This is quite rational given that the use of radiopharmaceuticals in neurodegenerative disorders is a new aspect, and numerous studies have been conducted in order to pass into clinical trials. The high number of novel radiopharmaceuticals for the direct and indirect imaging of neurodegenerative processes and their underlying pathologies can improve the diagnostic pathways. Several radiopharmaceuticals have been approved and are used in clinical routines for early and differential diagnoses, as well as for the evaluation of disease progression. Others are appreciated as valuable research tracers. However, as the more pathological components of different neurodegenerative diseases are discovered, new and interesting issues occur. Such issues include (i) the classification of neurodegenerative disorders in clinical routines, (ii) the identification of targets for possible, new radiotracers, (iii) the identification of novel radiotracer targets, and (iv) the accurate monitoring strategy of such therapy trials.

The environment of pharmacological discovery and therapy for AD has altered, notwithstanding the low success rate of AD medication development. Combination therapy is to be expected as the molecular understanding of AD biology and its adaptation in treatment advances. Better trial designs and demographics boost the likelihood of successful medication development. The clinical development of symptomatic therapies (STs) and disease-modifying therapeutics (DMTs) for Parkinson’s disease has significantly advanced, despite COVID-19 posing a significant obstacle in 2021–2022. It is nevertheless concerning that DMTs do not advance from Phase 2 to 3. HD-treatment possibilities are increasing, at present, and are more exciting thanks to drugs that target mHtt DNA and RNA. Similar to other neurodegenerative illnesses, HD has altered gut flora, and treating it may enhance treatment outcomes. Clinical trials should include standardized and validated rating scales, robust statistical analyses, and strategies to reduce placebo and bias effects.

Although there are numerous ongoing studies targeting the treatment of neurodegenerative disorders, we still do not have a cure. What is really missing is that scientists have not yet been able to recognize what leads to neurodegeneration. Is it pathogens, toxins, or daily activities? For years, the medical residency of neurology was very challenging since neurological diseases were not easily diagnosed and treatments were not available. To date, there is a considerable advancement in diagnosis since many inventions, such as PET/MRI, have been extensively used in the medical field. Moreover, it is believed that the use of monoclonal antibodies, genes, and peptides, which have presented interesting results for other incurable diseases, i.e., cancer, could be applied in neurodegenerative disorders. Although, for many years, the diagnosis of neurological diseases was complex and based on clinical manifestations, there are so many available diagnostic tools at present, and the research being conducted in labs for the identification of pathophysiology mechanisms can make us optimistic that this field will continue to advance. Additionally, scientists working in the drug delivery field should focus on the development of multifunctional carriers able to transport the active molecules to the targeted tissues. Moreover, combinations of antibodies, genes, or peptides should also be investigated in order to identify possible, better treatments.

As a result, in addition to delivering biological molecules to the brain via nanovehicles, novel administration routes should be investigated further in order to propose different ways of delivering biological molecules that bypass complex structures, such as the BBB and CFSB, and target the CNS. However, therapeutic and diagnostic options can be expanded if a critical understanding of the pathophysiological mechanism of neurodegeneration is achieved, which would allow for better treatment strategies and provide new opportunities for the management of such devastating diseases.

## Figures and Tables

**Table 1:** Pharmaceutical formulations with proteins and genes targeting neurodegenerative disorders using polymeric nanoparticles.

Active Molecule	Formulation	Targeting Disease	Method	Ref.
Vitamin-D-binding Protein	Poly(D,L-lactic acid-co-glycolic acid) (PLGA) nanoparticles	AD	In vivo 5XFAD mice	[101]
Basic fibroblast growth factor (bFGF)	Lecithin-modified polyethylene glycol-polylactide-polyglycolide (PEG-PLGA) nanoparticles	AD	In vivo male Sprague Dawley (SD) rats	[102]
Nerve growth factors (NGFs)	Poly(butly cyanoacrylate) (PBCA) nanoparticles	PD	In vivo male C57BL/6 mice	[103]
Ng receptor-siRNA and brain-derived neurotrophic factor (BDNF)	Crosslinked starch nanoparticles	AD	In vivo male SD mice	[104]
CGN QSH	PEGylated Poly(2-(N,N-dimethylamino) ethyl methacrylate) (PEG-PDMAEMA)	AD	In vivo transgenic AD mice	[105]
NGF, acteoside, and pDNA	Chitosan poly ethyleneglycol-poly lactic acid (PEG-PLA) nanoparticle	PD	In vivo female C57 mice	[106]
Rabies virus glycoprotein (RVG) peptide	Exosome curcumin/phenobornic acid-poly(2-(dimethylmino)ethyl acrylate) nanoparticle/siRNA	PD	In vivo C57BL/6 mice	[107]
miRNA-124-loaded RVG29	Polymeric nanoparticles	PD	In vivo mice	[108]
Epigallocatechin-3-gallate, β-site amyloid precursor protein (APP)-cleaving enzyme 1 antisense (BACE1-AS)	Pegylated PLGA nanoparticles	AD	In vivo APP/PS1 mice	[109]
Fusion peptide TPL	Pegylated PLGA nanoparticles	AD	In vivo mice	[110]
TGN and QSH	Pegylated PLA	AD	In vivo AD mice	[111]
Urocortin peptide	Pegylated PLA	PD	In vivo mice	[112]
H102 peptide	Pegylated PLA	AD	In vivo	[113]
Peptide sequence of Lys-Leu-Val-Phe-Phe (KLVFF)	poly ethylene glycol nanoparticles	AD	In vitro	[114]
Plasmid DNA-encoding BACE1-AS shRNA	PEGylated dendrigraft poly-l-lysines (DGLs)	AD	In vivo C57BL/6J mice/APP/PS1 double transgenic AD	[115]
miRNA-124	PLGA nanoparticles	PD	In vivo 6-hydroxydopamine (6-OHDA) mice	[116]
Cholesterol	PLGA nanoparticles	HD	In vivo mice	[117]
siRNA	Polyethylenimine nanoparticles	PD	In vivo Thy1-aSyn mice	[118]
siRNA, DNA	Chitosan-mangafodipir nanoparticles	ND	In vivo Tg GFP+ mice	[119]
siRNA	Chitosan	HD	In vivo YAC128 mouse	[120]

**Table 3:** Pharmaceutical formulations with proteins and genes targeting neurodegenerative disorders using other nanosystems.

Active Molecule	Formulation	Targeting Disease	Method	Ref.
NGF	Porous silicon nanostructures	Neuroprotection	In vivo	[136]
Plasmid containing α-synuclein RNAi	Oleic acid-coated iron oxide nanoparticles	PD	In vivo male C57BL/6 mice	[137]
pDNA	Gold nanoparticles	PD	In vivo male C57BL/6 mice	[138]
THRPPMWSPVWP peptide sequence	Gold nanoparticles	AD	In vivo Male Sprague rats	[139]
TPM (tetrapeptide from maize)	Gold nanoparticles	AD	In vivo female KM mice	[140]
Plasmid DNA	Gold nanoparticles	PD	In vivo C57bl/6 mice	[141]
D1 and Ang2 peptides	Gold nanorods	AD	In vivo Caenorhabditis elegans AD model	[142]
NGF	Poly(N-isopropylacrylamide), poly(L-lactic acid), and poly(L-lysine) dendrons	AD	In vitro	[143]
siRNA	RGV-Cyclodextrin nanoparticles	HD	In vitro	[144]

**Table 4:** The crucial questions for radiopharmaceuticals.

Questions (Radiopharmaceutical Applications)	Therapeutic Antibody or Specialized Radiopharmaceuticals
**Where the antibody goes?** (Biodistribution)	Therapeutic antibody
**Where is the target protein expressed?** (Assess expression and heterogeneity of target in tumor and normal tissues)	Radiopharmaceuticals
**Does the therapeutic antibody reach the target?** (Target engagement and dose range)	Therapeutic antibody
**Does therapeutic-antibody target interaction result in the expected biological effects?** (Response prediction/theranostic applications)	Therapeutic antibody
**Is there a specific pharmacodynamic marker linked to response?** (Imaging-surrogate efficacy endpoint)	Radiopharmaceuticals

**Table 5:** Radiopharmaceuticals used in AD, PD, and HD [168,169,170,171,172].

Radiopharmaceutical	Target	Disease	Method
[^11^C] Thioflavin-T analog	Aβ-PET	AD	In vivo Transgenic mice [168]
[^18^F] Florbetaben, [^18^F] Fluorodeoxyglucose	Aβ-PET	AD	Human trials [173,174], In vivo Transgenic mice [172]
[^18^F] Florbetapir	Aβ-PET	AD	In vitro [175], In vivo Transgenic mice [176]
[^18^F] Flutemetamol	Aβ-PET	AD	In vivo Wild-type Sprague Dawley rats and C57Bl/6N mice [169]
[^18^F] AZD4694, [^18^F]AD-269	Aβ-PET tracers	AD	Human trials [177], in vivo transgenic mice [178]
[^11^C] PBB3	Tau-PET tracers	AD	Human trials [179]
[^18^F] Flortaucipir (AV-1451)	Tau-PET tracers	AD	Human trials [180]
[^11^C] SIL5	α-syn-PET tracers	PD	In vivo male Sprague Dawley rats [181]
[^125^I] SIL23	α-syn-PET tracers	PD	Human brain tissues [182]
[^11^C] Raclopride	D2 receptors	HD	Human trials [183]
[^11^C] FLB 457	D2 receptors	HD	Human trials [183]

## Data Availability

Not applicable.
